# Reviewer acknowledgement 2013

**DOI:** 10.1186/1471-2342-14-3

**Published:** 2014-01-22

**Authors:** Fernando Marques

**Affiliations:** 1 BioMed Central, 236 Gray's Inn Road, WC1X 8HB, London, United Kingdom

## Abstract

**Contributing reviewers:**

The Editors of *BMC Medical Imaging* would like to thank all our reviewers who have contributed to the journal in Volume 13 (2013)

## 

Ahmed Abdel Razek

Egypt

Mohamed Abou El-Ghar

Egypt

Rodney Adam

US

Hiroyuki Akai

Japan

Banu Alicioglu

Turkey

Giovanni donato Aquaro

Italy

Majid Assadi

Iran

Adrian Barbu

US

Hamid Behnam

Iran

Nacim Betrouni

France

Mythreyi Bhargavan

US

Andrew Bierhals

US

Tiago Bilhim

Portugal

Michael Bouvet

US

Adrian Boyle

UK

Jian-guo Cao

China

Barry Cense

Japan

Kraisorn Chaisaowong

Germany

Dev Chakraborty

US

Phillipo Leo Chalya

Tanzania

Wenan Chen

US

Kyoung Im Cho

Korea, South

Rosario Cianci

Italy

Sarada Dakua

India

Sushmita Datta

US

Frederik De Keyzer

Belgium

Giorgio De Nunzio

Italy

Andrew Degnan

US

Bart Depreitere

Belgium

Anne Dompmartin

France

Sebastian Domsch

Germany

Francescamaria Donati

Italy

Matthias Eiber

Germany

Anders Eklund

US

Ayman El-Baz

US

Ravindhra Elluru

US

James Elmore

US

Jan Engvall

Sweden

Osman Erogul

Turkey

Gokhan Ertas

Turkey

Hannu Eskola

Finland

Gianluca Faggioli

Italy

Francesco Faita

Italy

Andriy Fedorov

US

Arne Fischmann

Switzerland

Timothy Fitzgerald

US

John Forder

US

Valentina Garibotto

Switzerland

Pantelis Georgiadis

Greece

Panagiotas GEORGOULIAS

Greece

Raúl González-García

Spain

Simon Graham

Canada

Christian Gueldner

Germany

Bob Guenther

US

Lan-Yuen Guo

Taiwan

Lothar Häberle

Germany

Jun Hatazawa

Japan

Daichi Hayashi

US

Marc Hickeson

Canada

Atsushi Hiraoka

Japan

Nina Hirata

Brazil

Philip Hodnett

Ireland

Maurits Jansen

UK

Soo Ji

US

Nadjia Kachenoura

France

Rathachai Kaewlai

Thailand

Takeshi Kamitani

Japan

Helen Keen

Australia

Hassan Kharita

Syria

Rami Korhonen

Finland

Noriyoshi Kuzushita

Japan

Choonsik Lee

US

Du Lianfang

China

Ezequiel Lopez-Rubio

Spain

Frank Ludwig

Germany

Peter Lundberg

Sweden

Baoming Luo

China

Pedro Machado

Portugal

S. Sara Mahdavi

Canada

Sanjay Mallya

US

Anne Martel

Canada

Hitoshi Masuda

Japan

Knut Matre

Norway

Grant Mawston

New Zealand

Martina McAteer

UK

Carmel Moran

UK

Arrate Muñoz-Barrutia

Spain

Raja Muthupillai

US

Frederico Neves

Brazil

Jaakko Niinimäki

Finland

Julie O'Brien

Ireland

Satoshi Okayama

Japan

Mehmet Ruhi Onur

Turkey

Ana-Maria Oros-Peusquens

Germany

Shanmugavadivu Pichay

India

Valeria Panebianco

Italy

Weijun peng

China

Antonino Perino

Italy

Stephen Pitts

US

James Pope

Australia

Emilio Puentedura

US

Li Ping Qi

China

Mario Rango

Italy

Alexander Rauscher

Canada

Francisco Ridocci

Spain

Francis Ring

UK

Giles Roditi

UK

Jan D. Rompe

Germany

Marek Ruchala

Poland

Balasrinivasa Sajja

US

Susumu Sato

Japan

David Schaeur

US

Vera Schrauwen-Hinderling

Netherlands

Neil Seligman

US

Renato Seracchioli

Italy

Kuniaki Seyama

Japan

Sarabjeet Singh

US

Jens Sommer

Germany

Jacob Sosna

Israel

Vivek Srinivasan

US

Oliver Stachs

Germany

Nili Steinberg

Israel

Jennifer Stone

Australia

Roland Syha

Germany

Etsuo Takada

Japan

Cher Heng Tan

Singapore

Sabina Tangaro

Italy

Mehmet Taskaynatan

Turkey

Catalina Tobon Gomez

Spain

Giorgio Treglia

Switzerland

Du-Yih Tsai

Japan

Panagiotis Tsiamyrtzis

Greece

Kenan Turgutalp

Turkey

Lindsay Turnbull

UK

Martin Ugander

Sweden

Stig Urheim

Norway

Thierry Van den Bosch

Belgium

Berit Verbist

Netherlands

Mathijs Versteylen

Netherlands

Petros Vlastarakos

UK

Olga Vriz

Italy

Tomaz Vrtovec

Slovenia

Steve Vucic

Australia

Jan Weis

Sweden

Andrew Whitelaw

UK

Steve Williams

UK

Alexander Wong

Canada

Jie Wu

US

Yihong Yang

US

Waleed Yousef

Egypt

E.A. Zanaty

Saudi Arabia

Chiara Zuiani

Italy

## Pre-publication history

The pre-publication history for this paper can be accessed here:

http://www.biomedcentral.com/1471-2342/14/3/prepub

